# Supplementary material for: Convergent pairs of highly transcribed genes restrict chromatin looping in Dictyostelium discoideum
Source: Nucleic Acids Res. 2025 Jan 22;53(2):gkaf006. doi: 10.1093/nar/gkaf006 (PMC11754127; doi:10.1093/nar/gkaf006)
Supplement: gkaf006_Supplemental_Files [file gkaf006_supplemental_files.zip › Zhegalova_Supplementary Materials_20-11-24_REV1_clean.docx]

**SUPPLEMENTARY MATERIALS**

**Supplementary Table S1.** Sequencing statistics of Hi-C libraries.

**Supplementary Table S2.** Las-loops identified in the vegetative (A) and late aggregation stages (B).

**Supplementary Table S3.** Loop coordinates in vegetative cells, migrating, and aggregating Dicty cells. The following parameters are listed: coordinates of the 2-kb genomic bin identified as the 5’-anchor (chrom1, start1, end1), coordinates of the 2-kb genomic bin identified as the 3’-anchor (chrom2, start2, end2), loop score (the same as the loop strength), loop type (elongated or regular), development stage.

**Supplementary Table S4.** Gene expression levels in vegetative, migrating, and aggregating Dicty cells, separately for replicates.

**Supplementary Table S5.** Loops containing genes with highly similar networks of gene ontology terms, as revealed by the best match average (BMA) analysis (BMA-loops). The following parameters are listed: loop coordinates (chrom, start, end), fold change over the random control, log10 of Mann-Whitney *p*-value, Kruskal-Wallis *p*-value; median BMA-value for all gene in loops, key GO terms.

**Supplementary Table S6.** Convergent (A) and divergent (B) gene pairs in the Dicty genome. The following parameters are listed for CGP: chrom, start, end of CGP; start and end of 5’-gene, start and end of 3’-gene, gene IDs, expression levels in TPM, length of genes, anchor group based on loop anchors intersection with CGP intergenic, log10 of intergenic length and sum gene length, group based on length both of the intergenic and gene length. The following parameters are listed for DGP: chrom, start, end of DGP; start and end of 5’-gene, start and end of 3’-gene, gene IDs, expression levels in TPM, intergenic length, expression group based on expression levels of both genes, anchor group based on loop anchors intersection with DGP intergenic,

**Supplementary Table S7.** Enhancer-like elements (ELEs) in the Dicty genome. Intergenic ELEs are designated by empty cell in the Gene ID column. The following parameters are listed: ELE coordinates (chrom, start, end), ID of the encompassing gene.

**Supplementary Table S8.** Dicty cohesin subunits: gene name, Dicty gene IDs, Uniprot ID, protein existence evidence from UniProt, and TPM-normalized transcription levels at four development stages. Median expression levels for all Dicty genes and beta-tubulin is also shown for comparison purpose.

**Supplementary Table S9.** Assessment of the RNA-polymerase density on Dicty chromatin and expected average initiation rate at TSS. Number of polymerases per kb of a gene is assumed based on yeast studies (1).

**Supplementary Table S10.** Preliminary optimization of parameters. The following parameters are listed: extruder processivity, extruder/polymerase speed ratio, transcription initiation rate, contact radius and loop strength.

**Supplementary Table S11.** Local optimization of parameters. The following parameters are listed: extruder processivity, extruder/polymerase speed ratio, transcription initiation rate, contact radius, average Pearson and Spearman correlations.

**Supplementary Table S12.** Whole-genome parameter optimization. The following parameters are listed: extruder processivity, extruder/polymerase speed ratio, transcription initiation rate, contact radius, region coordinates, average Pearson and Spearman correlations.


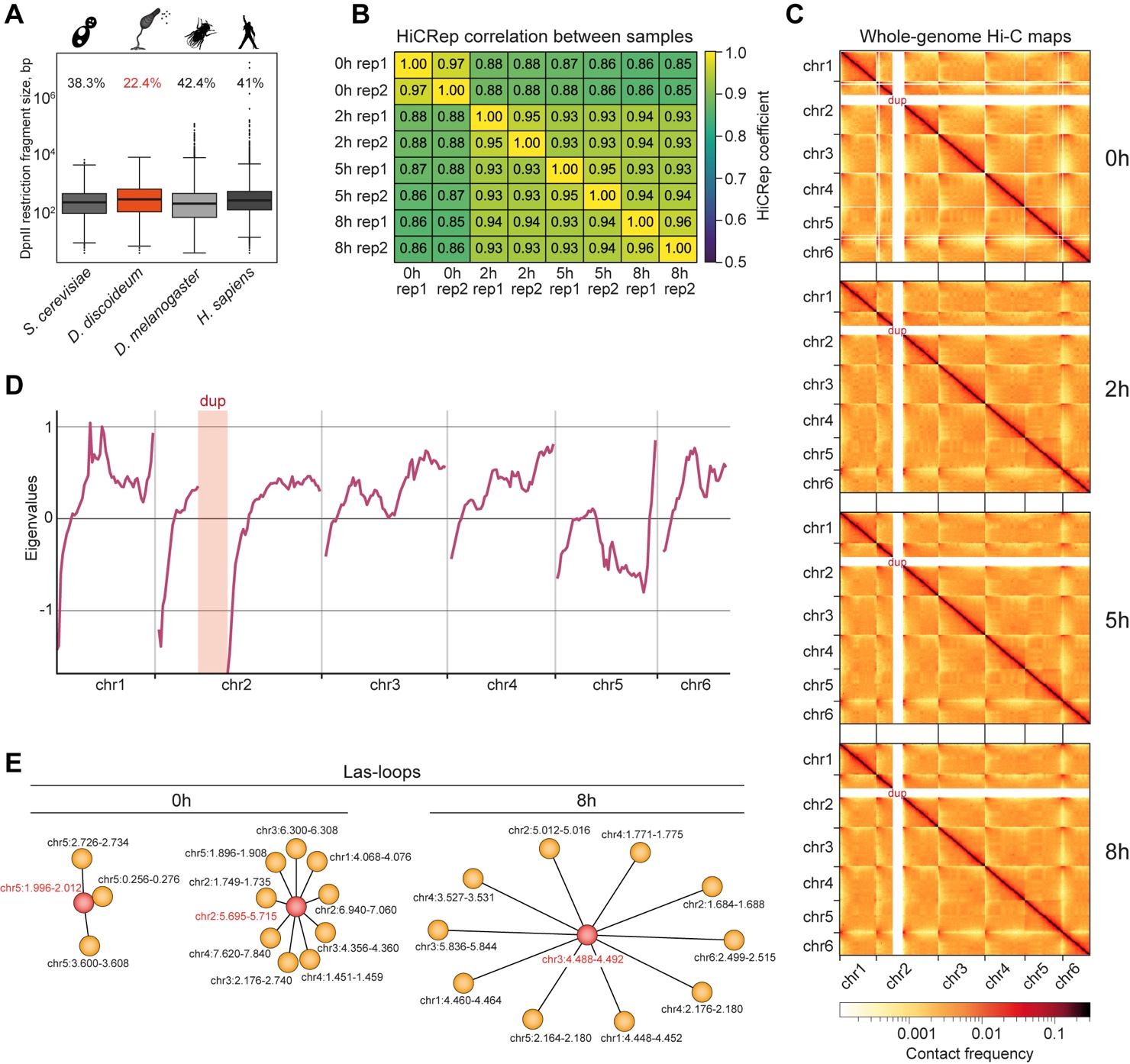


**Supplementary Figure S1.** (**A**) Size distribution of DpnII restriction fragments in *D. discoideum*, *S. cerevisiae*, *D. melanogaster*, and *H. sapiens* genomes. GC-content of the genome is shown above the boxplots. (**B**) Clustering of Hi-C replicates. (**C**) Whole-genome Hi-C maps at Dicty development stages. (**D**) Eigenvector profile across Dicty chromosomes. **e**, Network of las-loops identified in vegetative cells (0h) and late aggregates (8h).


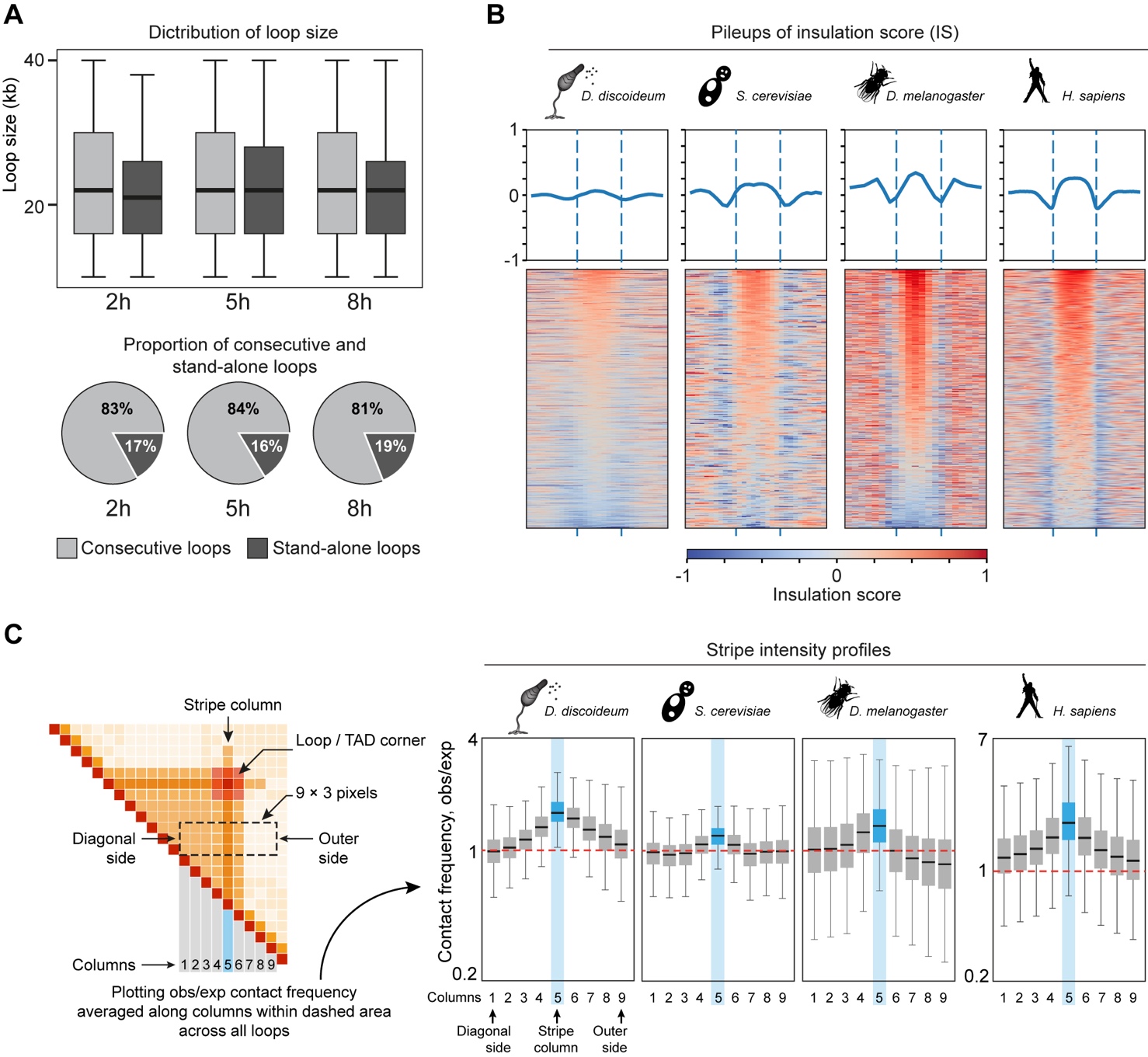


**Supplementary Figure S2.** (**A**) Size distribution (upper panel) and proportion (bottom panel) of consecutive and stand-alone loops. (**B**) Pileups of IS profiles (related to Figure 2D). Data source: *S. cerevisiae* - Micro-C XL in S-phase cells (51); *D. melanogaster* - Hi-C in spermatogonia (52); *H. sapiens* - Hi-C in GM12878 lymphoblastoid cells (53). (**C**) Schematic of stripe intensity quantification (left) and stripe intensity profiles derived from loops (Dicty, yeast, human cells) and TADs (Drosophila cells).


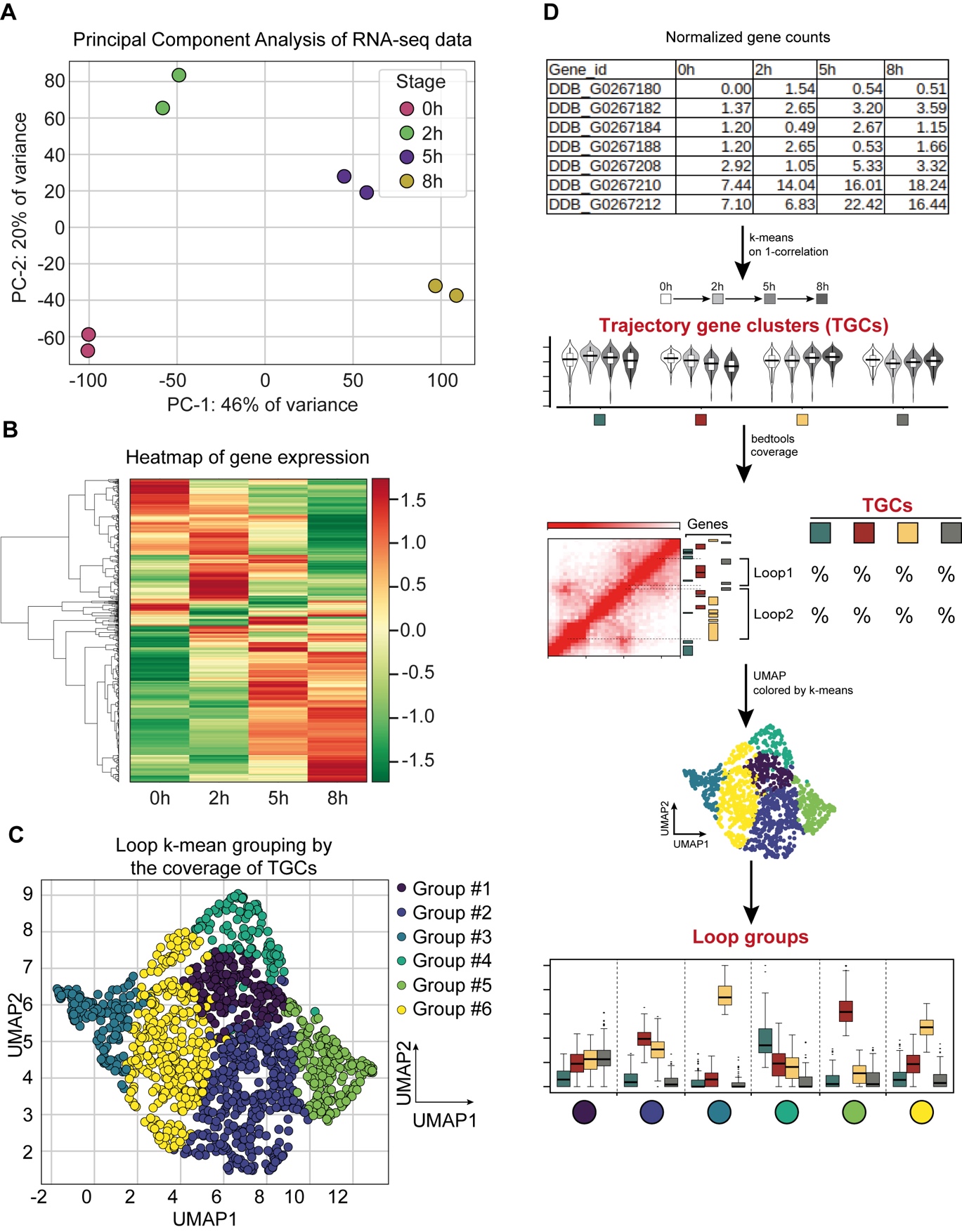


**Supplementary Figure S3.** (**A**) Clustering of RNA-seq replicates. (**B**) A heatmap of gene expression at Dicty development stages. One row represents one gene. Genes are ordered according to hierarchical clustering. (**C**) UMAP of loop k-means clustering according to the coverage of genes with the same developmental trajectories. (**D**) Schematic of the genes clustering into TGCs and grouping of loops by the TGC coverage.


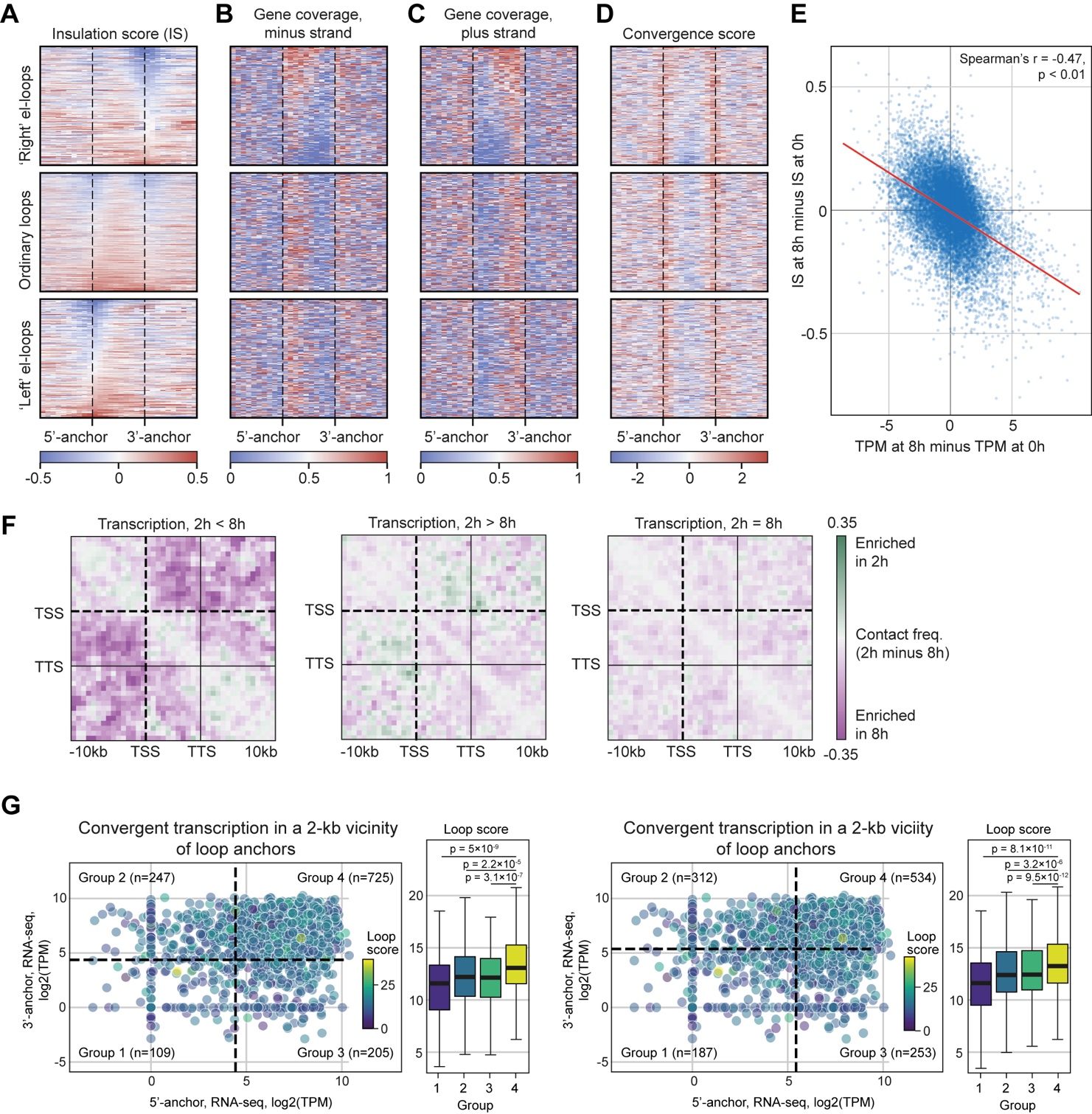


**Supplementary Figure S4.** (**A-D**) Pileups for Figures 4D-F. (**E**) Dependency between changes of insulation score (IS) and transcription in 2-kb bins in comparison between vegetative cells (0h) and late aggregates (8h). (**F**) Averaged Hi-C maps centered at genes downregulated (left panel) and upregulated (middle panel) at 2h stage compared to 8h stage, and at genes ubiquitously expressed at both stages (right panel). (**G**) Related to Figure 5A: the same picture, but groups of loops are defined by 25^th^ percentiles (left) and 35^h^ percentiles (dashed lines) of transcription level around loop anchors to demonstrate the robustness of the analysis.


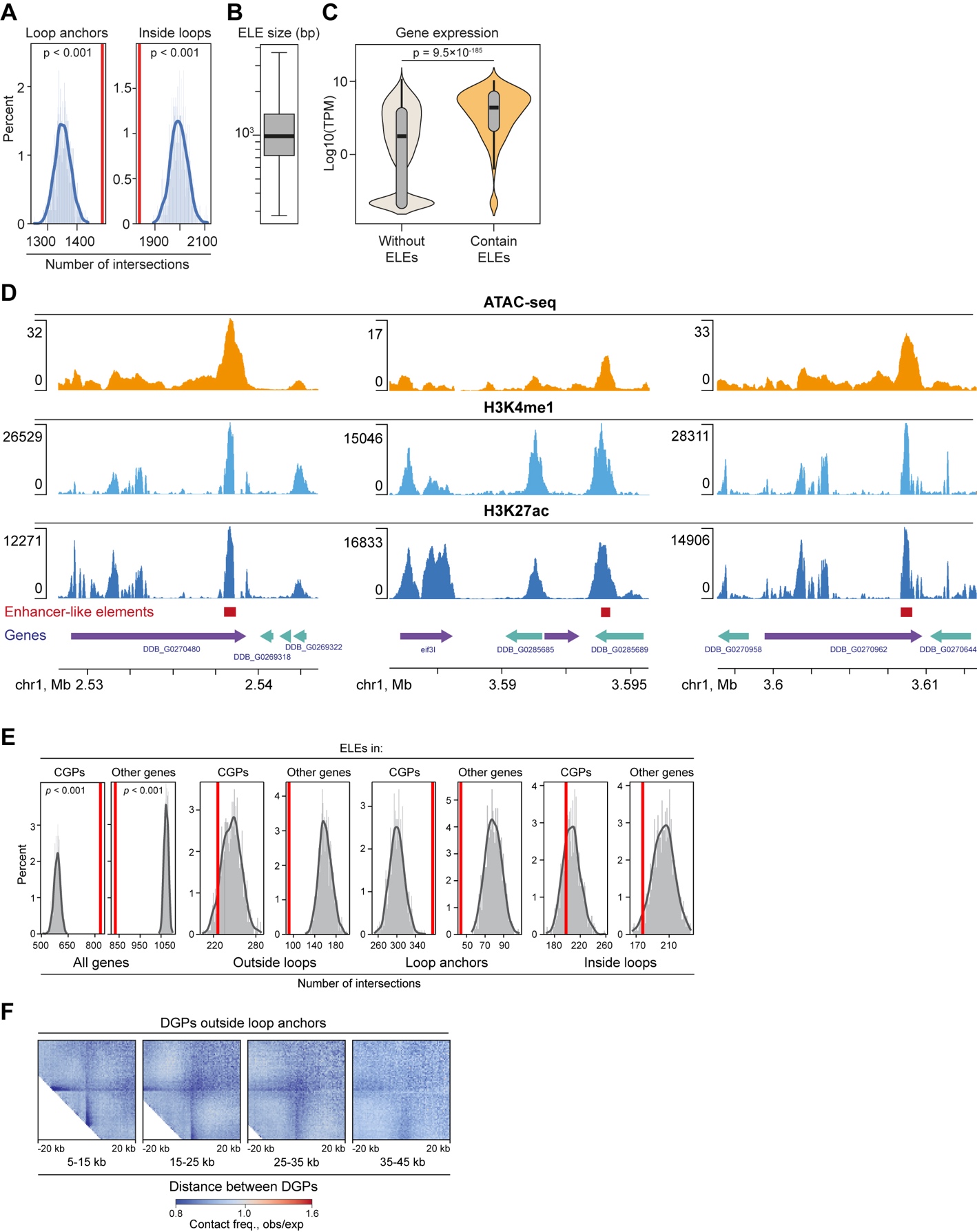


**Supplementary Figure S5.** (**A**) CGP enrichment at loop anchors and inside loops. (**B**) Size distribution of ELEs. (**C**) Distributions of expression level of genes which contain and do not contain ELEs. *p*-value in the MWU. (**D**) Representative examples of ELEs. H3K27ac, H3K4me1, ATAC-seq profiles and gene positions are shown. (**E**) Enrichment of ELEs in gene groups shown in Figure 5A. p-value in the permutation test. (**F**) Averaged interaction between DGPs separated by different genomic distances.


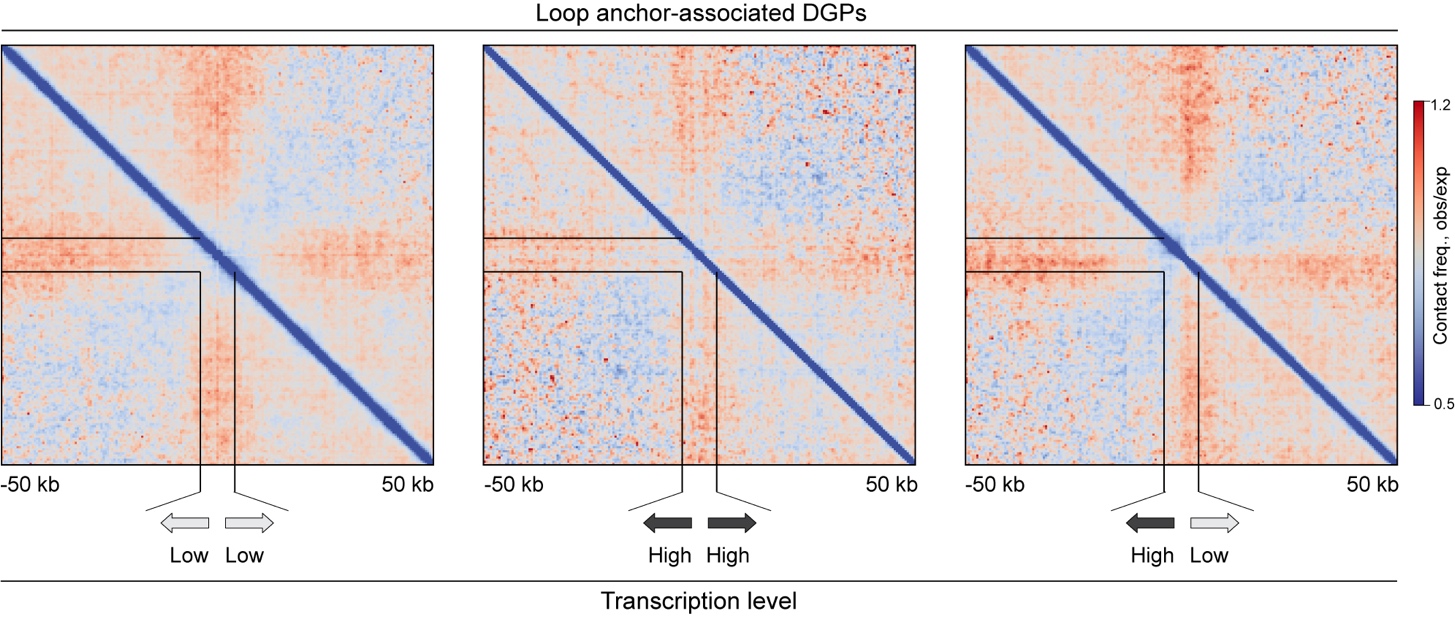


**Supplementary Figure S6.** Averaged Hi-C maps centered at divergent gene pairs (DGPs) with different levels of transcription (genes transcribed at high and low levels are highlighted with dark gray and light gray, respectively).


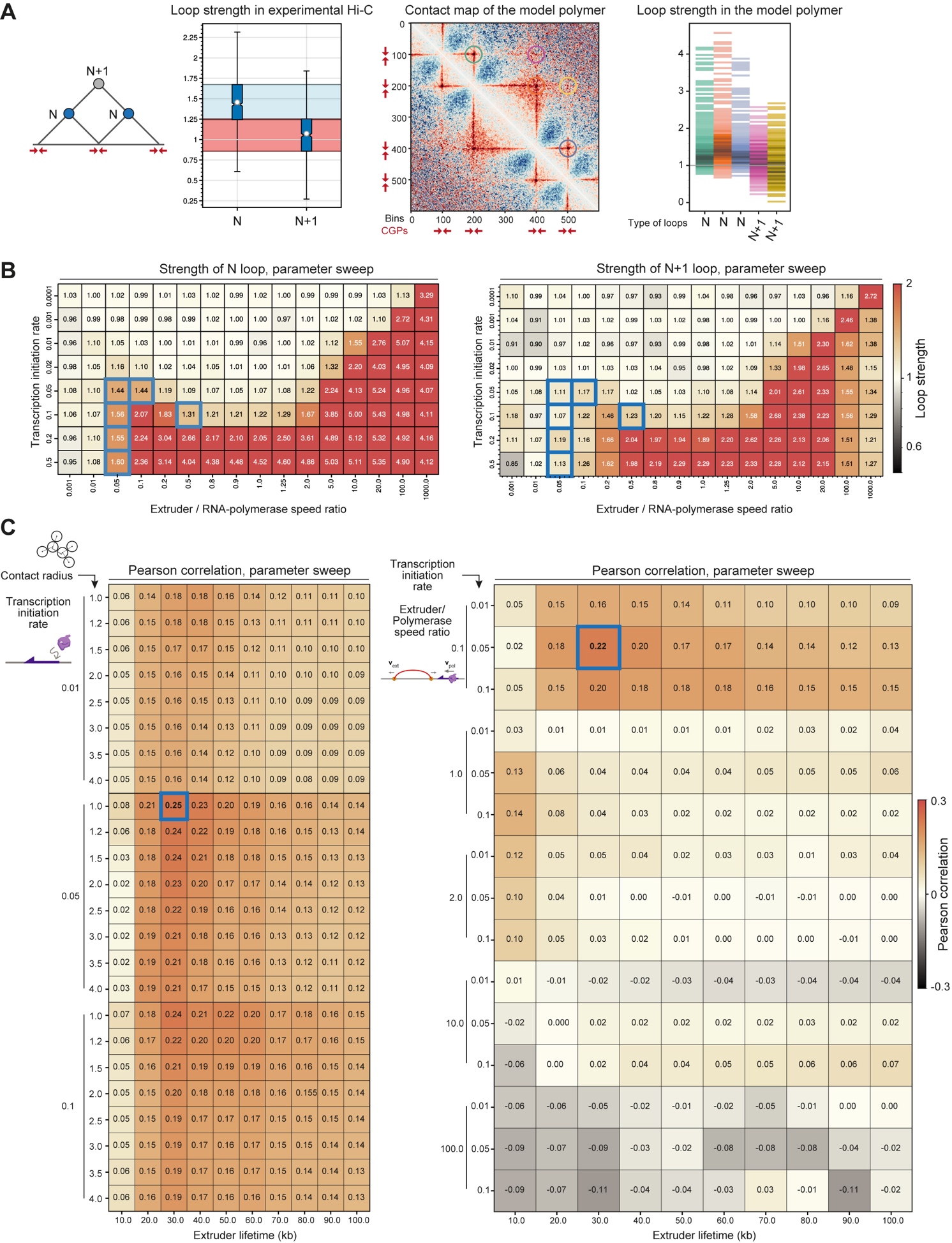


**Supplementary Figure S7.** Preliminary sweep and local optimization of polymer simulation parameters. (**A**) Quality metric for the preliminary parameters sweep. As Dicty loops are non-hierarchical, we used the median value of N loop strength from the experimental data, while keeping N+1 loop strength at around 1. Schematic of N and N+1 hierarchical loops (leftmost), loop strength in experimental Hi-C data (central left; blue zone - target values for the strength of N loops, red zone - target values for the strength of N+1 loops), model polymer used for preliminary parameter sweep (central right), loop strength distributions in the model polymer under different parameters (rightmost). Circles in different colors highlight loops in the model polymer used as a quality measurement, red arrows represent convergent gene pairs (CGPs). (**B**) Transcription initiation rate vs extruder/RNA-polymerase speed ratio preliminary parameter sweep using strength of average N loop (left) and strength of average N+1 loop (right) as target values for optimal parameter windows selection. Optimal values falling into the inter-quartile range of Hi-C loop strength are designated with blue rectangles. (**C**) Local parameter optimization using random genomic locus (chr4:750,000-1,000,000). Pearson correlation between experimental and simulated Hi-C maps is a quality metric for simulation performance. Transcription initiation rate and contact radius vs extruder lifetime in kb (left), extruder/RNA-polymerase speed ratio and transcription initiation rate vs extruder lifetime in kb (right). Optimal values are designated with blue rectangles.

**
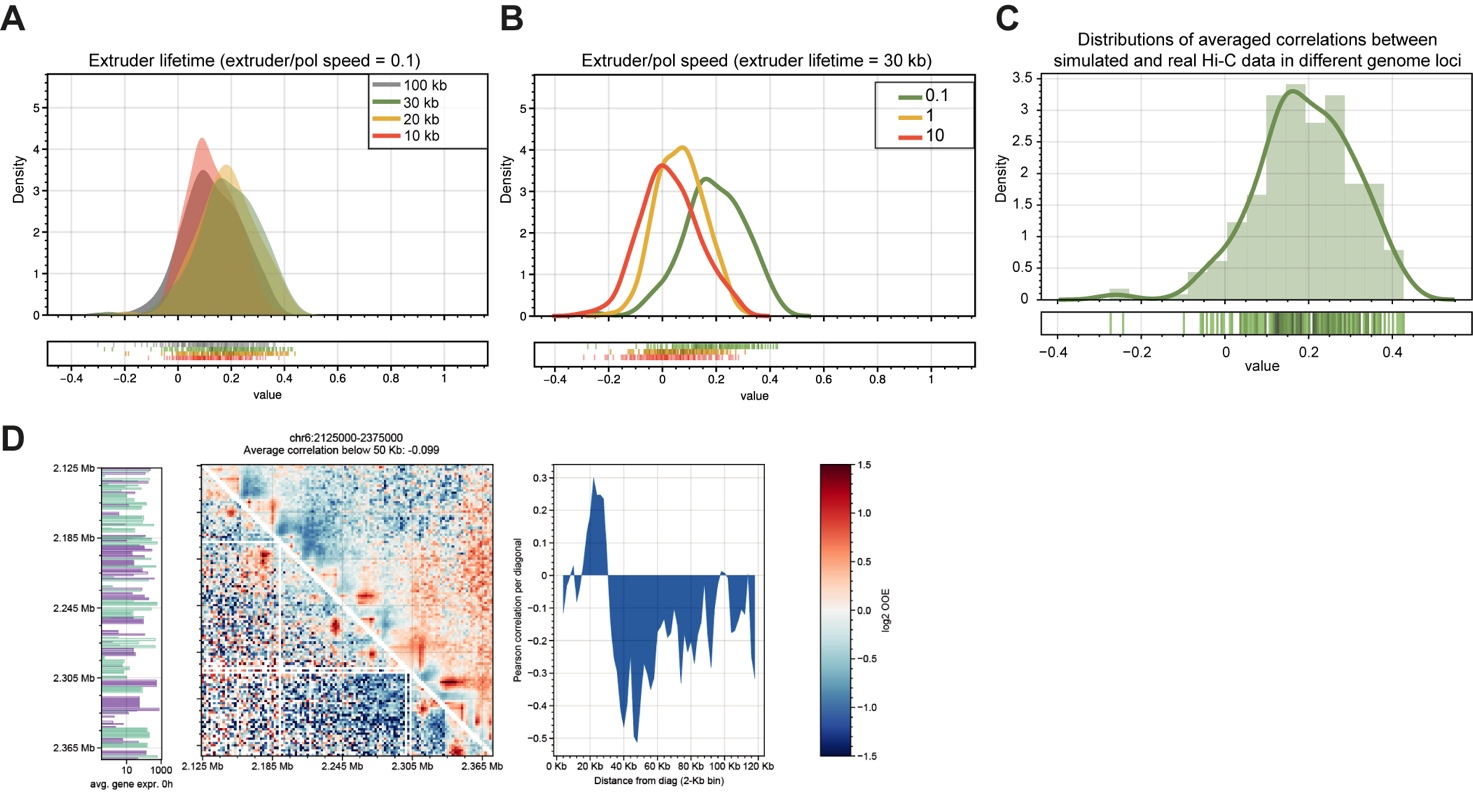
**

**Supplementary Figure S8.** Distributions of correlations between simulated and experimental Hi-C data averaged over 244 partially overlapping 250-kb intervals covering the entire Dicty genome. (**A**) Simulations performed at the extruder/polymerase speed ratio equal to 0.1, extruder lifetimes 10, 20 and 30 kb are tested. (**B**) Simulations performed at the extruder lifetime 30 kb, extruder/polymerase speed ratios equal to 0.1, 1 and 10 are tested. (**C**) Distribution of averaged correlations obtained at optimal simulation parameters: extruder/polymerase speed ratio = 0.1, extruder lifetime = 30 kb, transcription initiation rate = 0.05, contact radius = 1. Correlation has been calculated for contacts less or equal to 50 kb. (**D**) An example of the genome locus whose structure is poorly predicted by simulations.


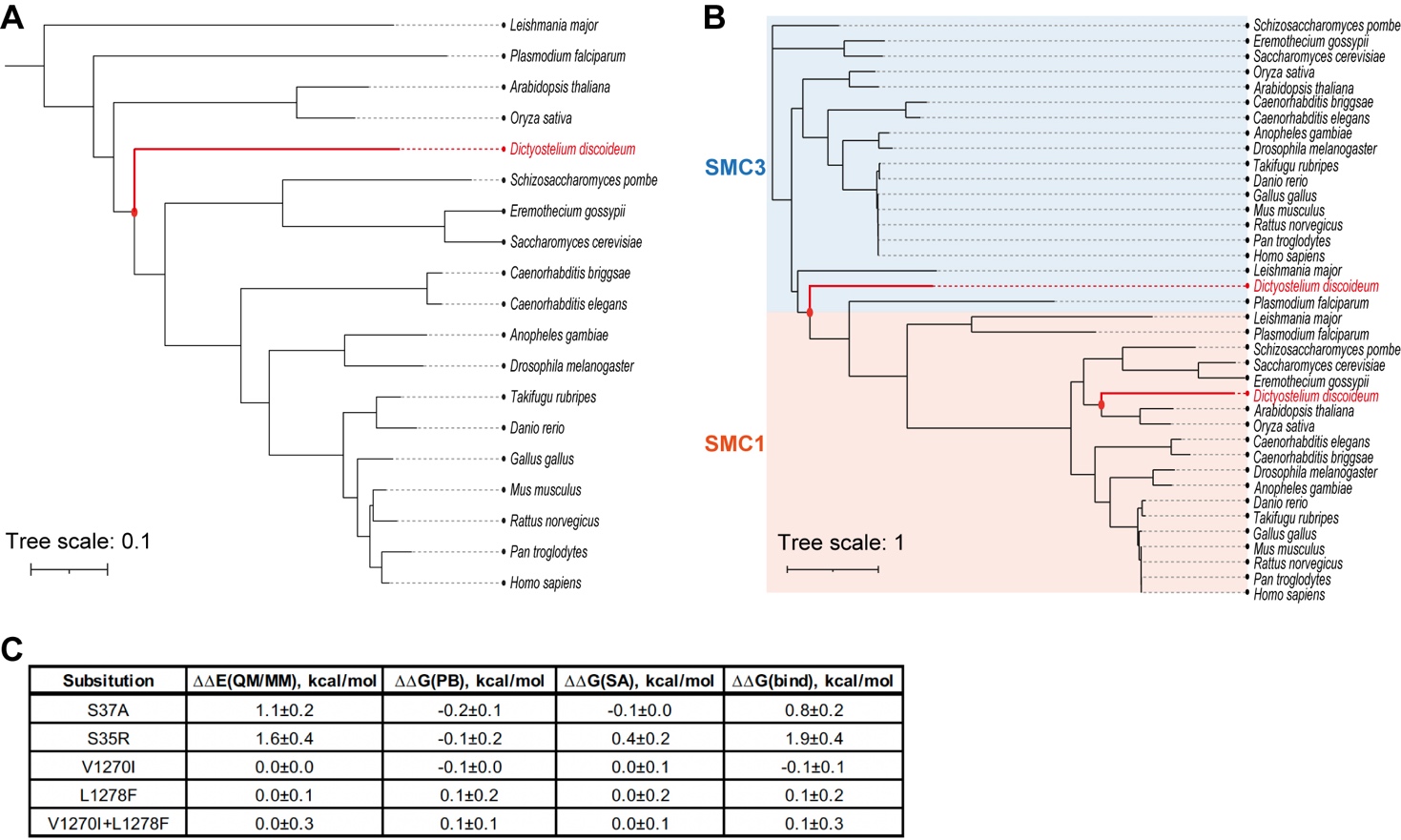


**Supplementary Figure S9.** Dicty cohesin substitutions. (**A**) Tree of Life from iTOL, Dicty is colored in red. (**B**) Phylogenetic tree of SMC3 and SMC1. SMC1 is highlighted in pink, SMC3 is highlighted in blue, Dicty is colored in red. (**C**) Resulting values in molecular dynamics modelling, columns: Dicty substitution, gas-phase QM/MM energy difference change, polar solvation energy difference change computed by solving Poisson-Boltzmann equation, non-polar solvation energy change calculated in terms of SASA/mbondi2 model.


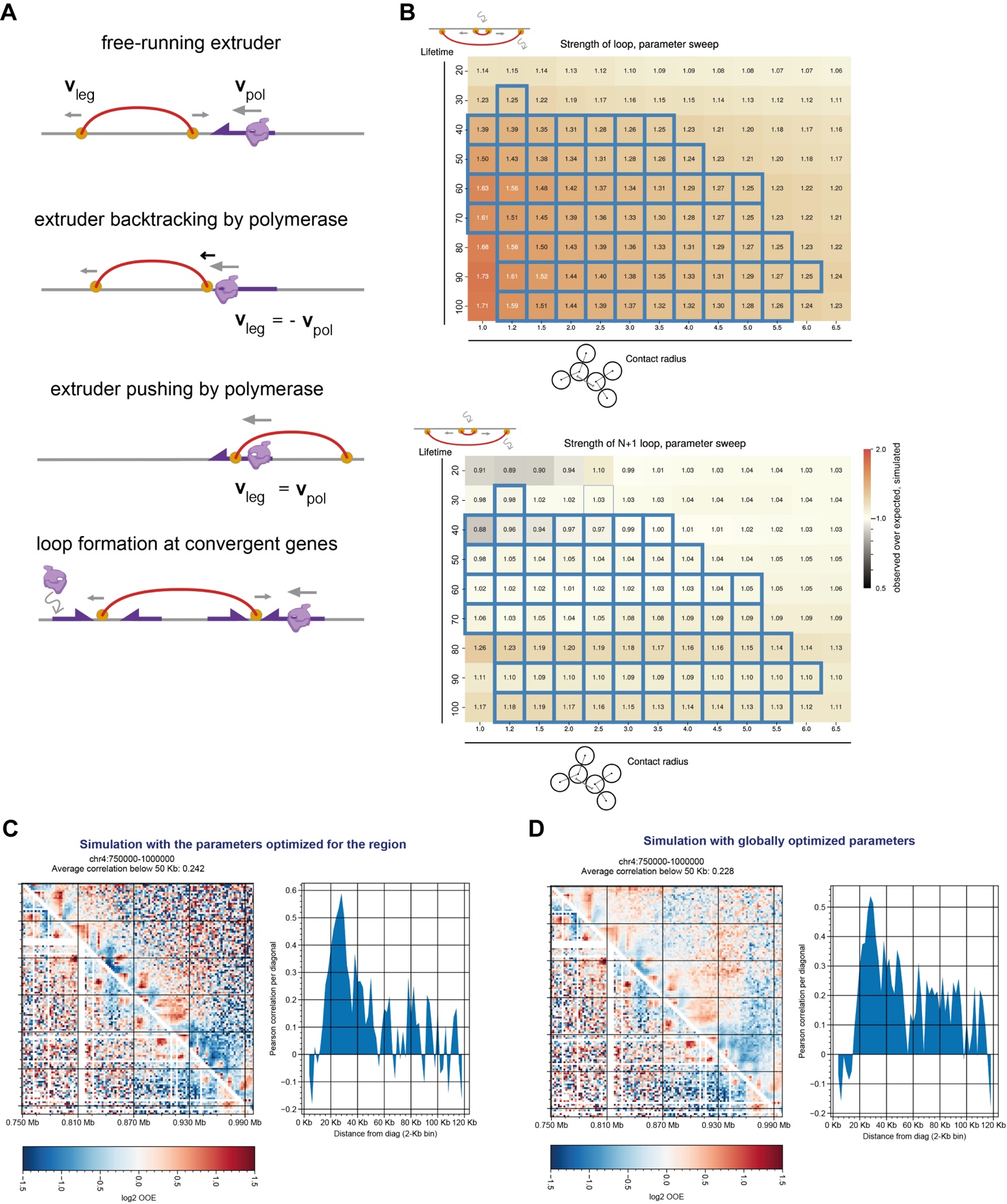


**Supplementary Figure S10.** (**A**), Different rules for 1D extrusion interplay with polymerase movement. (**B**), Lifetime vs contact radius cutoff for preliminary parameter sweep using strength of average N loop (left) and strength of average N+1 loop (right) as target values for optimal parameter windows selection. Optimal values falling into the interquartile range of Hi-C loop strength are designated with blue rectangles. (**C**), Representative example of simulated Hi-C data (upper map) in comparison with experimental Hi-C data (bottom map) for a locus simulated with optimal parameters in Supplementary Figure S7C. Distributions of the Pearson correlation coefficient averaged over different genomic distances are shown to the right of the maps. (**D**), Same locus as in Supplementary Figure 10C, but simulated with globally optimized parameters.
